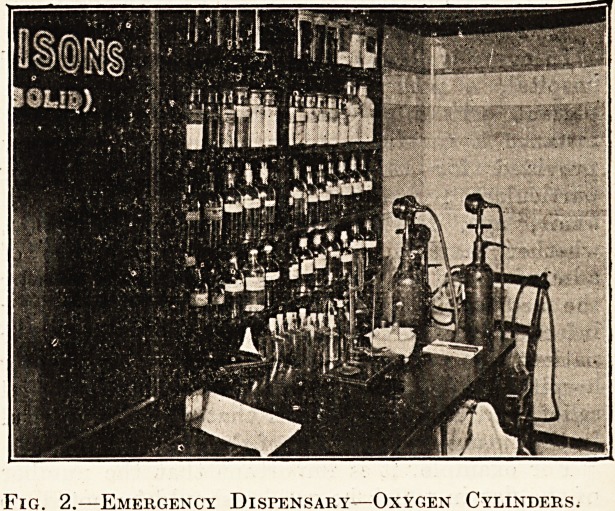# The Admission Department of a General Hospital

**Published:** 1907-04-27

**Authors:** 


### HOSPITAL ADMINISTRATION.

### CONSTRUCTION AND ECONOMICS.

### THE ADMISSION DEPARTMENT OF A GENERAL HOSPITAL.

II. ITS THOROUGH SUPERVISION.

(*Concluded.*)

In every well-appointed and properly managed hospital, a porter will be at hand when the patient arrives at the entrance. In the first instance a careful note is made, on the forms provided for this purpose, of the following particulars regarding the patient: Date, full name, age, full address, occupation, religion, whether married or single, the exact time of admission and the time when the patient is seen by the medical officer, whether there is, or is not, any infectious disease in or near his residence, whether patient has previously visited that or any other hospital, whether patient is in receipt of parochial relief, and any other detail that might be useful in the future management of the case.

For example, it is important that the number of the flat in which he resides should be noted, so that in the event of any urgent message being conveyed (say by night) to his friends there may be no difficulty in finding the exact location of the dwelling. This applies more particularly to Scottish hospitals. While a porter is obtaining all the details from such relatives or friends as may accompany the patient, the medical officer will have been summoned. The following is a copy of the form:—

Date,
Name, Med. or Surg.
Age, Country, Religion, Soc. Cond.
Occupation,
Residence,
Infectious disease in or near Residence,
Previous visits to this or other Hospitals,
Does Patient receive Parochial Relief?
Subscriber's Letter or Doctor's Recommendation,
Other Letter,
Entered by Time,
Examined by Time,
Result,

Every patient seeking admission should be carefully examined by the medical officer on duty for the day, and, as already stated, no patient should be refused admission until he is seen by the medical superintendent or his deputy.

Should the medical officers on duty consider any patient unsuitable, they will fill in the following form for transmission to the medical superintendent and await his decision:—

We hereby certify that we have examined
and do not consider that he is a fit patient for admission into the Infirmary as
Resident Assistant.
Supt. Resident Assistant.

It then rests with the medical superintendent or his deputy to see the patient and give a final decision.

If the case is a suitable one, the porter will receive instructions to convey the patient direct to the ward to which he is assigned, and in male cases he may be required to assist in undressing him. This he should do with as little inconvenience to the patient as possible. If there is no special urgency, the medical officer gives instructions for the attendant to remove the patient to the bathing department, where he is stripped and bathed. His clothes are treated in a disinfector, if necessary, and are thereafter stored till his discharge, or are sent home with any relative who may accompany him. After the patient is bathed, he should be wrapped in hospital garments, covered with blankets and removed to the ward. A careful note should be made of all money or valuables in the patient's possession, and such valuables should be handed over for lodgment in the safe. Special care should be exercised in the bathing of children and helpless patients.

The nearest relative present should accompany the patient to the ward and be allowed to remain till the house surgeon on duty has obtained a history and full details of the case. Before leaving the hospital the relatives should be furnished with a visiting card giving the visiting days and hours and the rules to be observed in visiting patients. Any correspondence which accompanies the patient from his medical adviser should be handed at once to the medical officer on duty, and should accompany the patient to the ward in order that the house physician or surgeon under whose care the patient is placed may have the fullest information available. Neglect in the observance of this simple rule may cause much confusion and delay in the subsequent treatment of the case.

If there is any suspicion that the patient suffers from any infectious or contagious disease, he should be retained in the examination-room till after consultation with the medical superintendent. If the case proves infectious, the room should be closed, the sanitary authorities notified, and the patient removed to a hospital for infectious disease at the earliest possible moment. Before this room is used again it should be thoroughly disinfected and washed down. The walls of these consulting-rooms being tiled, this can be expeditiously and inexpensively accomplished.

When any case of poisoning is admitted, the house physician should deal with it on the spot. An "emergency outfit" containing stomach-pump, antidotes, and everything which might be required, should be fitted up in a sealed cupboard in a small emergency dispensary, etc., near the admission department. The object of the seal is that the dispenser may see every morning whether the apparatus and contents have been used, in order that he may put in a fresh supply, so that no possible emergency can occur without the fresh remedies being at hand. It is always advantageous to have a description of the usual antidotes for use in case of poisoning in this same cupboard. Figs. 1 and 2 illustrate such an emergency dispensary as referred to, with cupboard containing antidotes and serums, also cylinders of oxygen and emergency drugs.

If a criminal case or an attempted suicide is admitted, the medical officer on duty should notify the police authorities at once, unless there are special reasons for omitting this procedure.

When a patient is admitted seriously ill and there is immediate danger to life, the relatives should be informed without delay.

### Keeping Records.

The following records should be kept in the admission department:—

1. A general register, giving the date, number of patient in this journal, name, number in surgical or medical journal, age, whether married or single, full address, recommender (employer or doctor), accident or emergency, whether from out-patient department, nature of ailment (whether medical, surgical, gynæcological, etc.), a note of the medical officer who examines the patient, and the ward to which the patient is sent. A column should be reserved for "remarks." This journal should contain a record of every patient who seeks advice or admission, whether treated or not, as separate admission journals must be kept for the in-patient and the out-patient departments.

Fig. 1.—Emergency Dispensary—Antidote Cupboard.

2. A bed-book, giving each day the number of patients in each ward in the hospital, dividing the males from females, noting the number admitted, dismissed, and died. The same book should contain a record of the name, age, and number of the ward of each patient admitted and dismissed, giving at the foot a summary of the total number admitted to each department, and the total number of patients in the hospital each day.

Fig. 2.—Emergency Dispensary—Oxygen Cylinders.

3. A medical and surgical register, giving the date, number on the general register, number of ward, age, country, whether married or single, occupation, full address, recommender or medical adviser, number of days the patient has been in the hospital, nature of disease or injury, date of dismissal, and result; and a space should be reserved for "remarks," such as cause of death, reason for dismissal, whether patient was sent to convalescent home or received assistance, such as from the Samaritan Fund, etc.

4. An abstract-book, giving a summary of these details in order that the number of patients in the hospital can be compared from day to day, month to month, and year to year.

5. In addition to these records, an alphabetical index of every patient admitted to hospital should be kept up to date and available for reference, giving the patient's name, the number of the ward, and the date of admission and dismissal.

---

The Town Clerk of Paddington has informed the various Borough Councils of the Metropolis that the proposed conference on the Prevention of Pulmonary Tuberculosis has been postponed from May 1 to May 6.

The following changes, we understand, are under consideration at St. Bartholomew's Hospital: in the gynæcological department the staff is to be strengthened by the appointment of an assistant physician accoucheur; while in the aural department a new appointment, that of assistant aural surgeon, is to be created. The hospital is also to be fitted with a thorough internal telephonic exchange system. It is pleasant to record that the present management of this our oldest hospital in London is so actively keeping in touch with modern advances.

## Figures and Tables

**Fig. 1. f1:**
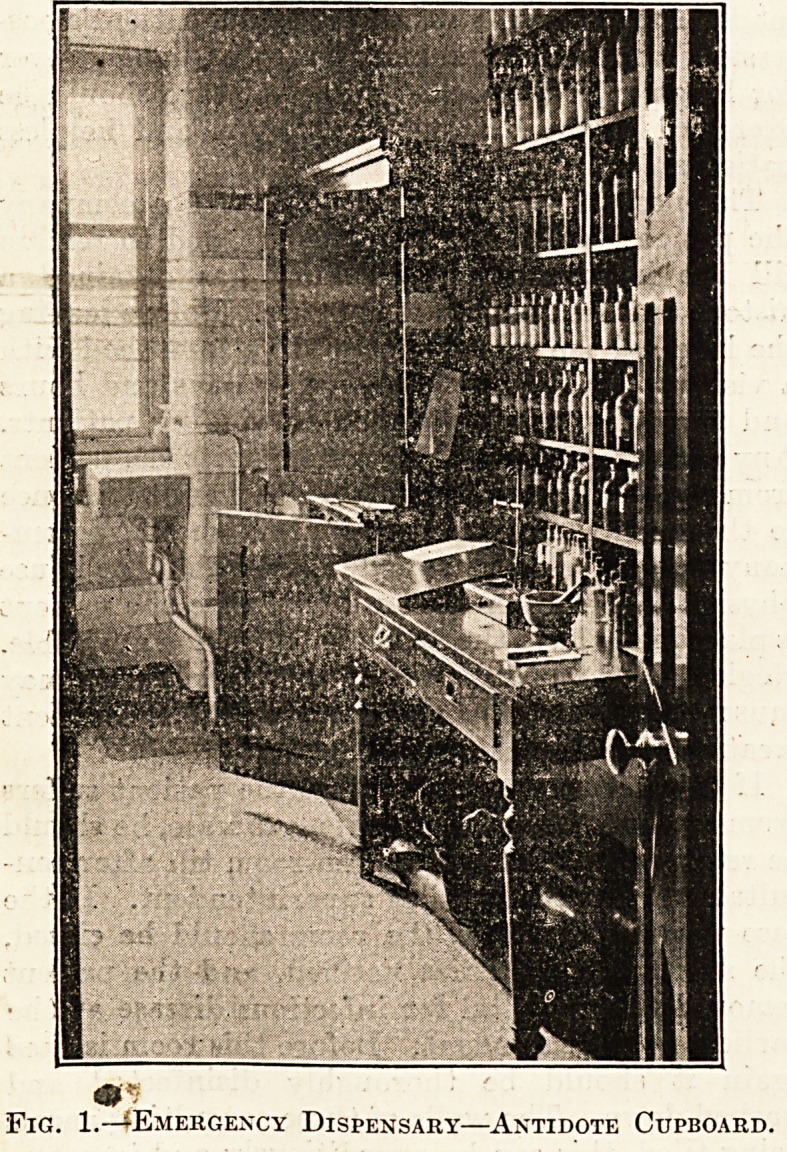


**Fig. 2. f2:**